# Prognostic Role of Neutrophil to Lymphocyte Ratio in COVID-19 Patients: Still Valid in Patients That Had Started Therapy?

**DOI:** 10.3389/fpubh.2021.664108

**Published:** 2021-06-15

**Authors:** Monica Gelzo, Sara Cacciapuoti, Biagio Pinchera, Annunziata De Rosa, Gustavo Cernera, Filippo Scialò, Mauro Mormile, Gabriella Fabbrocini, Roberto Parrella, Ivan Gentile, Giuseppe Castaldo

**Affiliations:** ^1^CEINGE-Biotecnologie Avanzate Scarl, Naples, Italy; ^2^Dipartimento di Medicina Molecolare e Biotecnologie Mediche, Università di Napoli Federico II, Naples, Italy; ^3^Dipartimento di Medicina Clinica e Chirurgia, Università di Napoli Federico II, Naples, Italy; ^4^Divisione di Malattie Infettive Respiratorie, Dipartimento di Malattie Infettive e Emergenze Infettive, Ospedale Cotugno, AORN dei Colli, Naples, Italy; ^5^Dipartimento di Medicina Traslazionale, Università della Campania L. Vanvitelli, Naples, Italy

**Keywords:** COVID-19, neutrophil to lymphocyte ratio, interleukin-6, myeloperoxidase, corticosteroid therapy

## Abstract

COVID-19 may appear with a widely heterogeneous clinical expression. Thus, predictive markers of the outcome/progression are of paramount relevance. The neutrophil/lymphocyte ratio (NLR) has been suggested as a good predictive marker of disease severity and mortality. Accordingly, we found that NLR significantly increased in parallel with the WHO severity stage in COVID-19 patients during the I^st^ wave (March-May 2020; *n* = 49), due to the significant reduction of lymphocyte and the significant increase of neutrophil in severe COVID-19 patients. While, we did not observe significant differences of NLR between the WHO severity stage among COVID-19 patients of the II^nd^ wave (September 2020-April 2021; *n* = 242). In these patients, the number of lymphocytes and neutrophils did not change significantly between patients of different severity subgroups. This difference likely depends on the steroids therapy that the patients of the II^nd^ wave performed before hospitalization while most patients of the I^st^ wave were hospitalized soon after diagnosis. This is also confirmed by serum interleukin (IL)-6 and myeloperoxidase (MPO) that gradually increased with the disease stage in patients of the I^st^ wave, while such biomarkers (whose production is inhibited by steroids) did not show differences among patients of the II^nd^ wave in different stages. Thus, the NLR could be tested at diagnosis in naïve patients before starting therapies.

## Introduction

The Coronavirus disease 2019 (COVID-19) may appear with a heterogeneous clinical expression, i.e., from asymptomatic or mild to severe forms causing a significant loss of lives. Therefore, it is fundamental to early identify COVID-19 patients with a higher risk of a poor clinical outcome and predictive markers are of paramount relevance (1, 2).

Various studies concluded that the neutrophil/lymphocyte ratio (NLR) has a good predictive value on disease severity and mortality in patients with COVID-19 infection (3). On the other hand, the NLR ratio is an easy and rapid prognostic marker in a myriad of clinical conditions that include solid tumors (4), chronic obstructive pulmonary disease (5), liver cirrhosis (6), rheumatoid arthritis (7), acute pancreatitis (8), sepsis (9) and psoriasis (10).

COVID-19 pandemic had a I^st^ wave in Italy between March and May 2020. During this period, we studied 49 patients, including all consecutive patients admitted to our hospitals with a diagnosis of COVID-19 (11). After a lockdown during summer 2020, since September 2020 the pandemic in Italy had a II^nd^ wave (12) and we studied 242 further patients. Herein, we report a comparison of some laboratory parameters including NLR in COVID-19 between the patients of the I^st^ and II^nd^ waves.

## Methods

We enrolled adult patients with a diagnosis of COVID-19 (SARS-CoV-2 infection) admitted from March to May 2020 (I^st^ wave) or from September 2020 to April 2021 (II^nd^ wave) at one of the following hospitals: Department of Clinical Medicine and Surgery - Section of Infectious Diseases, University Hospital Federico II, Naples; Department of Infectious Disease and Infectious Urgencies, Cotugno Hospital, AORN dei Colli, Naples. The study was approved by the Ethical Committee of the University Federico II of Naples; the lone exclusion criterion was the refusal or the impossibility to obtain the informed consent. No patient admitted to our hospitals was excluded.

The 49 patients of the I^st^ wave included 19 females (38.8%), and had a mean age of 59 years (range: 24–92 years). The frequencies of comorbidities in I^st^ wave patients were the following: hypertension, 40%; cardiovascular disease, 35%; chronic obstructive pulmonary disease, 25%; diabetes, 15%; chronic renal failure, 5%.

The 242 patients of the II^nd^ wave included 121 females (50.0%), and had a mean age of 52.9 years (range: 17–94 years). The frequencies of comorbidities in II^nd^ wave patients were the following: hypertension, 43%; cardiovascular disease, 20%; chronic obstructive pulmonary disease, 22%; diabetes, 13%; chronic renal failure, 6%.

The diagnosis of COVID-19 was confirmed by molecular analysis (RT-PCR) of the nasopharyngeal swab (11). All the enrolled patients were classified on the basis of the seven ordinal scale made by the World Health Organization (WHO)-Research and Development Blueprint expert group and used in previous influenza studies. According to this scale, patients with COVID-19 can be classified as: (1) not hospitalized with resumption of normal activities; (2) not hospitalized, but unable to resume normal activities; (3) hospitalized, not requiring supplemental oxygen; (4) hospitalized, requiring supplemental oxygen; (5) hospitalized, requiring nasal high-flow oxygen therapy, non-invasive mechanical ventilation, or both; (6) hospitalized, requiring extra corporeal membrane oxygenation, invasive mechanical ventilation, or both; and (7) death. For each patient we considered the worst WHO stage during the infection (13, 14). We divided our population study in three subgroups: WHO 3, WHO 4, and WHO 5–7 (including COVID-19 patients with WHO stage from 5 to 7). Whole blood samples were collected at admission in tubes containing EDTA and then immediately analyzed for neutrophil and lymphocyte count. Serum samples were separated from blood cells after the collection in tubes without anticoagulant and stored at −80°C until interleukin (IL)-6 and myeloperoxidase (MPO) measurements by Human Magnetic Luminex Assay on Biorad Bio-Plex 100 system (Labospace s.r.l., Milan, Italy).

Continuous data were reported as mean and standard error (SE). Comparisons between two groups were performed by Mann-Whitney *U*-test. Statistical differences between three groups were assessed by ANOVA test and Bonferroni test as *post-hoc* test. Categorical data were reported as frequency and percentage. The chi-square test was used to compare the frequency of categorical variables between groups. To test the association of neutrophils, lymphocytes and NLR values vs. age, steroids and azithromycin therapies, a linear regression analysis with neutrophils, lymphocytes and NLR as dependent variables was performed using a stepwise approach. Statistical analyses have been evaluated by SPSS (version 26, IBM SPSS Statistics). Graphics have been performed by KaleidaGraph software (version 4.5.4, Synergy, Reading, PA, USA). *P*-values < 0.05 were considered as significant.

## Results

**Figure 1:**
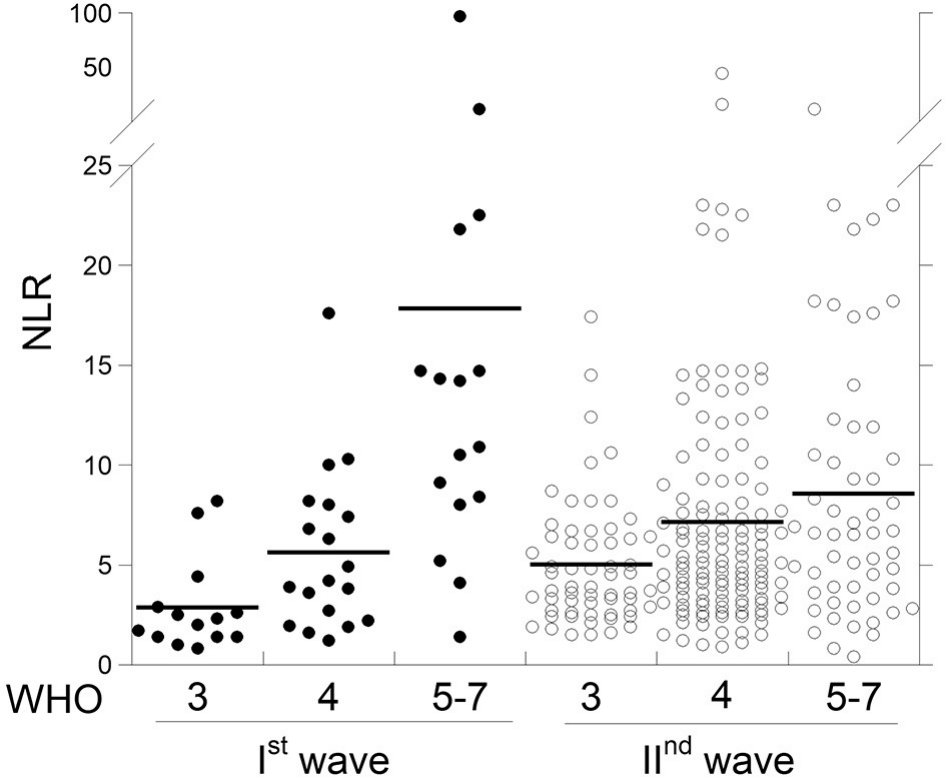
Scattergram of neutrophil/lymphocyte ratio (NLR) in COVID-19 patients from the two waves at different WHO stages.

Figure 1 shows the data of NLR in the 49 COVID-19 patients of the I^st^ wave and in the 242 COVID-19 patients of the II^nd^ wave, classified on the basis of the WHO score. Table 1 reports either the comparison of the data between the WHO subgroups of patients of the same wave, and the comparison of patients of the two waves bearing to the same WHO subgroup. Among the patients of the I^st^ wave, the NLR was significantly higher in WHO 5–7 than both WHO 3 and WHO 4 subgroups due to: (i) the number of lymphocytes that was significantly lower and the number of neutrophils that was higher in WHO 5–7 than both WHO 3 and WHO 4 patients. While, in patients of the II^nd^ wave the NLR values were not statistically different among the WHO subgroups. This depends on the number of either neutrophiles and lymphocytes that was not different among the WHO subgroups.

**Table 1:** Comparison of age and laboratory parameters in 49 COVID-19 patients of I^st^ wave and 242 patients of II^nd^ wave at admission with different severity according to worst WHO stage for each patient.

	**Wave**	**WHO 3**	**WHO 4**	**WHO 5–7**	**ANOVA**
*N*	I^st^	14	19	16	–
	II^nd^	60	127	55	–
Age (years)	I^st^	48.8 (3.5)	60.4 (3.5)^a^	66.3 (3.6)^b^	^*^
	II^nd^	38.9 (1.9)	56.7 (1.4)^a^	58.8 (2.0)^b^	^**^
	I^st^ vs. II^nd^	^*^	n.s.	n.s.	
IL-6 (pg/mL)	I^st^	168 (55)	399 (179)	703 (401)	n.s.
	II^nd^	38 (5)	60 (24)	41 (7)	n.s.
	I^st^ vs. II^nd^	^*^	^**^	^*^	
MPO (ng/mL)	I^st^	624 (118)	785 (71)	917 (83)^b^	n.s.
	II^nd^	389 (19)	322 (23)	350 (15)	n.s.
	I^st^ vs. II^nd^	^*^	^**^	^**^	
Neutrophils (N/mmc)	I^st^	4514 (560)	5255 (687)	9130 (1523)^b^	^*^
	II^nd^	5790 (324)	6332 (247)	7685 (426)^b^	n.s.
	I^st^ vs. II^nd^	n.s.	n.s.	n.s.	
Lymphocytes (N/mmc)	I^st^	2082 (284)	1118 (98)^a^	770 (169)^c^	^**^
	II^nd^	1425 (97)	1246 (63)	1229 (117)	n.s.
	I^st^ vs. II^nd^	^*^	n.s.	n.s.	
NLR	I^st^	2.9 (0.6)	5.6 (0.9)^a^	17.8 (5.5)^c^	^**^
	II^nd^	5.0 (0.4)	7.1 (0.6)	8.5 (1.0)	n.s.
	I^st^ vs. II^nd^	^*^	n.s.	^*^	

* *p < 0.01;*

** *p < 0.001.*

a *p < 0.01, WHO 4 vs. WHO 3.*

b *p < 0.01, WHO 5–7 vs. WHO 3.*

c *p < 0.01, WHO 5–7 vs. both WHO 3 and WHO 4.*

*NLR: neutrophil/lymphocyte ratio; n.s.: not significant. Mean and standard error (SE)*.

Furthermore, the values of IL-6 resulted higher in all 49 patients of the I^st^ wave, with an increase (although not significant) with the WHO stage, while in patients of the II^nd^ wave the values of IL-6 were significantly lower in each WHO subgroup as compared to the corresponding subgroup of patients of the I^st^ wave, with no differences between patients of different WHO stages. Similarly, the values of serum MPO resulted higher in all 49 patients of the I^st^ wave, with an increase (although not significant) with the WHO stage, while in patients of the II^nd^ wave the values of MPO were significantly lower in each WHO subgroup as compared to the corresponding subgroup of patients of the I^st^ wave, with no differences between patients of different WHO stages.

Among the II^nd^ wave, 155/242 patients performed steroids and/or azithromycin therapies before hospitalization differently from all patients of the I^st^ wave that were hospitalized soon after diagnosis. As reported in Table 2, a significant higher percentage of patients treated with both steroids and azithromycin was observed in WHO 4 and WHO 5–7 subgroups as compared to WHO 3. Therefore, we evaluated the associations of neutrophils, lymphocytes and NLR vs. the age, steroids and azithromycin therapies in each WHO subgroup (Table 3). Linear regression analysis revealed that only lymphocyte number was independently related to the age in WHO 3 and WHO 5–7 subgroups. In addition, we found that neutrophil number and NLR were positively related to steroids therapy in WHO 4 subgroup as well as lymphocytes in WHO 3. While, no significant relation was observed for azithromycin, as independent variable.

**Table 2:** Steroids and/or azithromycin therapies before hospitalization in II^nd^ wave patients.

**Subgroups**	**Not treated**	**Only steroids**	**Only azithromycin**	**Steroids and azithromycin**
WHO 3	48 (80)	5 (8)	1 (2)	6 (10)
WHO 4	27 (21)^a^	24 (19)	7 (6)	69 (54)^a^
WHO 5–7	12 (22)^a^	10 (18)	4 (7)	29 (53)^a^

a *p < 0.0001, vs. WHO 3. N (%)*.

**Table 3:** Linear regression analysis in II^nd^ wave COVID-19 patients.

	**Age**		**Steroids**		**Azithromycin**	
	**Slope**	***P*-value**	**Slope**	***P*-value**	**Slope**	***P*-value**
**Neutrophils**						
WHO 3	−0.209	0.055	0.201	0.062	0.180	0.085
WHO 4	0.014	0.438	0.160	**0.036**	0.050	0.287
WHO 5–7	0.105	0.219	0.196	0.072	0.009	0.475
**Lymphocytes**						
WHO 3	−0.225	**0.042**	0.249	**0.027**	0.213	0.051
WHO 4	−0.097	0.138	−0.005	0.479	−0.034	0.354
WHO 5–7	−0.313	**0.010**	−0.157	0.126	−0.163	0.117
**NLR**						
WHO 3	0.097	0.231	0.146	0.134	0.025	0.424
WHO 4	0.100	0.131	0.162	**0.034**	0.034	0.354
WHO 5–7	0.164	0.115	0.097	0.241	0.058	0.336

*Significant values are reported in bold*.

## Discussion

We found an increase of NLR values in 49 patients with COVID-19 of the I^st^ wave, with a significant increase of the index in parallel with the WHO disease severity, that depends on the gradual increase of neutrophils and on the gradual decrease of lymphocytes in WHO 5–7 patients (11). These evidences fully agree with previous studies (3), among which that of Qin et al. (15) which included more than 400 COVID patients. On the other hand, the cytokine storm that occurs in patients with severe COVID-19, confirmed by the high serum levels of IL-6 in our patients of the I^st^ wave, contributes to the lymphocyte exhaustion (11).

Surprisingly, when we analyzed the NLR in 242 patients with COVID-19 of the II^nd^ wave, we did not observe any difference of the ratio between patients of different WHO stage. Furthermore, we also lack to observe any difference in the number of lymphocytes and neutrophils between patients of different WHO stages. These differences between the patients of the two COVID-19 waves are not due to the younger age of the patients of the II^nd^ wave because the linear regression analysis showed that age did not significantly influence NLR and neutrophil within none of the three WHO subgroups although, a negative relation between lymphocytes number and age was observed in WHO 3 and WHO 5–7 subgroups. Thus, the differences between the two waves likely depend on the different therapies that the patients performed before hospitalization and thus before the sampling, as steroids therapy was the only independent variable related to NLR and neutrophils in the widest subgroup (WHO 4). In fact, all the 49 patients of the I^st^ wave were diagnosed as COVID-19 for symptoms followed by molecular analysis on nasopharyngeal swab (often completed 2 or 3 days after the sampling), and they were soon hospitalized after the result. Such patients were treated with antivirals that now we know to be less effective than hoped (12). While, most patients from the II^nd^ wave were diagnosed by molecular analysis performed when they were still asymptomatic, mostly because they had had a contact with a COVID patient and had been traced (12). In all cases, the result of the nasopharyngeal test was obtained within 1 day (thanks to the improvement of laboratory organization), and all the patients started to be treated several days before hospital admission. In our population study, 64% of patients of the II^nd^ wave assumed steroids (12) and/or azithromycin for several days before hospitalization despite the use of dexamethasone results in lower mortality only among COVID-19 patients who were receiving invasive mechanical ventilation (i.e., patients in advanced WHO stages) (16), while in patients not requiring respiratory support the immunosuppressive effects of glucocorticoids hamper antiviral responses (17).

However, the effects of such drugs observed in our patients of the II^nd^ wave, were: a slight reduction of neutrophil number, possibly due to a transient effect of steroids after a few days of therapy (18) combined to the same effect of azithromycin (19); a reduction of neutrophil activity due to steroids (20) demonstrated by the lower values of serum MPO; the inhibition of pro-inflammatory citokines among which IL-6; and a consequent less severe lymphocyte exhaustion.

A study limitation is represented by the relatively small number of patients of I^st^ wave in comparison to II^nd^ wave. Further studies need to confirm our findings as a recent work documents NLR predictive value on both I^st^ and II^nd^ waves (21). However, at the state of the art, there is not a clear consensus on the role of such drugs, frequently used friendly without medical supervision (22) and on their impact on COVID-19 patients. In any case, the present data suggest that such therapies impair the use of NLR as a marker of outcome and disease severity in COVID-19 patients, and its use should be limited to naïve patients before starting potential interfering therapies.

## Data Availability Statement

The raw data supporting the conclusions of this article will be made available by the authors, without undue reservation.

## Ethics Statement

The studies involving human participants were reviewed and approved by Ethical Committee of the University Federico II of Naples. The patients/participants provided their written informed consent to participate in this study.

## Author Contributions

GCa, GF, IG, and RP: design of the work, manuscript writing, and validation. MG, SC, BP, AD, GCe, FS, and MM: methodology, investigation, and data analysis. All authors read and approved the final manuscript.

## Conflict of Interest

The authors declare that the research was conducted in the absence of any commercial or financial relationships that could be construed as a potential conflict of interest.

## References

[1] Wu Z Mc Googan JM. Characteristics of and important lessons from the coronavirus disease 2019 (COVID-19) outbreak in China: summary of a report of 72 314 cases from the Chinese Center for Disease Control and Prevention. JAMA. (2020) 323:1239–42. 10.1001/jama.2020.2648 32091533

[2] Liang WH Liang HR Ou LM Chen B Chen A Li C. Development and validation of a clinical risk score to predict the occurrence of critical illness in hospitalized patients with COVID-19. JAMA Intern Med. (2020) 180:e202033. 10.1001/jamainternmed.2020.2033 32396163 PMC7218676

[3] Li X Liu C Mao Z Xiao M Wang L Qi S. Predictive values of neutrophil-to-lymphocyte ratio on disease severity and mortality in COVID-19 patients: a systematic review and meta-analysis. Crit Care. (2020) 24:647. 10.1186/s13054-020-03374-8 33198786 PMC7667659

[4] Templeton AJ McNamara MG Šeruga B Vera-Badillo FE Aneja P Ocaña A. Prognostic role of neutrophil-to-lymphocyte ratio in solid tumors: a systematic review and meta-analysis. J Natl Cancer Inst. (2014) 106:dju124. 10.1093/jnci/dju124 24875653

[5] Perrotta F Nigro E Pafundi PC Polito R Nucera F Scialò F. Adiponectin is associated with neutrophils to lymphocyte ratio in patients with chronic obstructive pulmonary disease. COPD. (2020) 18:70–5. 10.1080/15412555.2020.1857718 33302720

[6] Peng Y Li Y He Y Wei Q Xie Q Zhang L. The role of neutrophil to lymphocyte ratio for the assessment of liver fibrosis and cirrhosis: a systematic review. Expert Rev Gastroenterol Hepatol. (2018) 12:503–13. 10.1080/17474124.2018.1463158 29629626

[7] Erre GL Paliogiannis P Castagna F Mangoni AA Carru C Passiu G. Meta-analysis of neutrophil-to-lymphocyte and platelet-to-lymphocyte ratio in rheumatoid arthritis. Eur J Clin Invest. (2019) 49:e13037. 10.1111/eci.13037 30316204

[8] Kong W He Y Bao H Zhang W Wang X. Diagnostic value of neutrophil-lymphocyte ratio for predicting the severity of acute pancreatitis: a meta-analysis. Dis Markers. (2020) 2020:9731854. 10.1155/2020/9731854 32454909 PMC7232731

[9] Huang Z Fu Z Huang W Huang K. Prognostic value of neutrophil-to-lymphocyte ratio in sepsis: a meta-analysis. Am J Emerg Med. (2020) 38:641–7. 10.1016/j.ajem.2019.10.023 31785981

[10] Paliogiannis P Satta R Deligia G Farina G Bassu S Mangoni AA. Associations between the neutrophil-to-lymphocyte and the platelet-to-lymphocyte ratios and the presence and severity of psoriasis: a systematic review and meta-analysis. Clin Exp Med. (2019) 19:37–45. 10.1007/s10238-018-0538-x 30478648

[11] Cacciapuoti S De Rosa A Gelzo M Megna M Raia M Pinchera B. Immunocytometric analysis of COVID patients: a contribution to personalized therapy? Life Sci. (2020) 261:118355. 10.1016/j.lfs.2020.118355 32871183 PMC7456265

[12] Palmieri L Palmer K Lo Noce C Meli P Giuliano M Floridia M. Differences in the clinical characteristics of COVID-19 patients who died in hospital during different phases of the pandemic: national data from Italy. Aging Clin Exp Res. (2020) 33:193–9. 10.1007/s40520-020-01764-0 33345291 PMC7750107

[13] von Cube M Grodd M Wolkewitz M Hazard D Lambert J. Harmonizing heterogeneous endpoints in COVID-19 trials without loss of information-an essential step to facilitate decision making. MedRxiv [Preprint]. (2020). 10.1101/2020.03.31.20049007

[14] WHO Working Group on the Clinical Characterization and Management of COVID-19 infection. A minimal common outcome measure set for COVID-19 clinical research. Lancet Infect Dis. (2020) 20:e192–7. 10.1016/S1473-3099(20)30483-7 32539990 PMC7292605

[15] Qin C Zhou H Zhang S Yang S Tao Y Xie C. Dysregulation of immune response in patients with COVID-19 in Wuhan. China Clin Infect Dis. (2020) 71:762–8. 10.1093/cid/ciaa248 32161940 PMC7108125

[16] The RECOVERY Collaborative Group. Dexamethasone in hospitalized patients with COVID-19 – preliminary report. New Engl J Med. (2021) 384:693–704. 10.1056/NEJMoa2021436 32678530 PMC7383595

[17] Cain DW Cidlowski JA. After 62 years of regulating immunity, dexamethasone meets COVID-19. Nat Rev Immunol. (2020) 20:587–8. 10.1038/s41577-020-00421-x 32778829 PMC7416654

[18] Miseviciene V Liakaite G Kevalas R. Short course of systemic corticosteroids in wheezy children: still an open question. Adv Respir Med. (2019) 87:209–16. 10.5603/ARM.a2019.0035 31476008

[19] Zimmermann P Ziesenitz VC Curtis N Ritz N. The immunomodulatory effects of macrolides-a systematic review of the underlying mechanisms. Front Immunol. (2018) 9:302. 10.3389/fimmu.2018.00302 29593707 PMC5859047

[20] Ronchetti S Ricci E Migliorati G Gentili M Riccardi C. How glucocorticoids affect the neutrophil life. Int J Mol Sci. (2018) 19:4090. 10.3390/ijms19124090 PMC6321245 30563002

[21] Synolaki E Papadopoulos V Divolis G Tsahouridou O Gavriilidis E Loli G. The activin/Follistatin-axis is severely deregulated in COVID-19 and independently associated with in-hospital mortality. J Infect Dis. (2021) 223:1544–54. 10.1093/infdis/jiab108 33625513 PMC7928794

[22] Capoluongo ED Amato F Castaldo G. The friendly use of chloroquine in the COVID-19 disease: a warning for the G6PD-deficient males and for the unaware carriers of pathogenic alterations of the G6PD gene. Clin Chem Lab Med. (2020) 58:1162–4. 10.1515/cclm-2020-0442 32333649

